# A Data Transfer Fusion Method for Discriminating Similar Spectral Classes

**DOI:** 10.3390/s16111895

**Published:** 2016-11-14

**Authors:** Qingyan Wang, Junping Zhang

**Affiliations:** Harbin Institute of Technology, School of Electronics and Information Engineering, Harbin 150001, China; zhangjp@hit.edu.cn

**Keywords:** hyperspectral image, transfer learning, adaboost, fusion

## Abstract

Hyperspectral data provide new capabilities for discriminating spectrally similar classes, but such class signatures sometimes will be difficult to analyze. To incorporate reliable useful information could help, but at the same time, may also lead increased dimensionality of the feature vector making the hyperspectral data larger than expected. It is challenging to apply discriminative information from these training data to testing data that are not in the same feature space and with different data distributions. A data fusion method based on transfer learning is proposed, in which transfer learning is introduced into boosting algorithm, and other out-date data are used to instruct hyperspectral image classification. In order to validate the method, experiments are conducted on EO-1 Hyperion hyperspectral data and ROSIS hyperspectral data. Significant improvements have been achieved in terms of accuracy compared to the results generated by conventional classification approaches.

## 1. Introduction

With advanced sensors and space technology, now it is possible to access to remote sensing (RS) image data, which potentially provide more information individually and as a time series. RS has become an indispensable tool in many scientific disciplines. It is one of the major tools in monitoring our earth environment in a cost-effective way. Hyperspectral sensors simultaneously capture hundreds of narrow and contiguous spectral bands from a wide range of the electromagnetic spectrum. Due to their capability to precisely characterize the spectral signatures of different materials, hyperspectral images have been extensively used in the last decades in remote sensing applications. In such context, hyperspectral images are informative sources for detailed mapping, environmental monitoring, modeling, and biophysical characterization of agricultural crops [1,2,3,4].

Apart from deploying improved hardware system approaches [5], efficient application of this advanced capability should be with sophisticated hyperspectral image processing and analysis methods. While any of multispectral classification methods may be directly extended to hyperspectral images, there are additional challenges in the huge training data requirements, computational cost, and constraints on exploiting the information content. Classification of hyperspectral imagery is usually performed in a reduced feature space whose dimensionality is significantly lower than the number of original spectral bands [6].

Those will limit the direct application of multispectral image classification methods for hyperspectral image classification. Consequently, several image pre-processing techniques are now available for hyperspectral dimensionality reduction while using multispectral classification methods. Many other methods have also been introduced such as Spectral Angle Mapper (SAM), Spectral Feature Fitting, and Spectral Information Divergence [7], which are quite specific for hyperspectral image, learning-based artificial neural networks, and support vector machines [8,9,10].

Classification of a single hyperspectral image on a manifold, a feature space of reduced dimension developed via a nonlinear method, has been investigated in several works [11,12,13]. The trained classifiers are typically valid only for the corresponding remote sensing data set. For subsequent RS images over the same area, e.g., additional training samples are required, and the classifier must be retrained, for the variation of either signature or environmental conditions during the acquisition. It is still expected that two images are associated in some way in terms of class-dependent signatures and classification models. Although significant progress has been made in developing approaches in hyperspectral image classification, studies assessing the generalization and transfer characteristic of the spectral details of hyperspectral data for independent image classification are still limited. Exploring the association between data sets is an interesting and charming topic in the machine learning community and often referred to as transfer learning [14]. In addition, transfer learning can be used in the different domains or multi-task learning. On the basis, it is proposed a boosting algorithm to address inductive transfer learning problems, called TrAdaBoost [15]. TrAdaBoost has been efficiently used in text data mining. It is proposed a general instance weighting framework for domain adaptation to achieve instances transfer learning [16].

Manifold alignment (MA), where a joint manifold representing multiple images is obtained by aligning similar geometries, is a potentially attractive strategy for transfer learning from a geometric point of view [17,18,19]. A relevant development is the knowledge transfer or semi-supervised learning based classification methods [20,21,22,23,24,25]. In these methods, training data from one image are used for classifying another image of the same or adjacent area. Land cover classification accuracies from this approach are reported to be comparable with the image-based training data.

It is proposed an effective fusion method that makes use of the out-date image data. The main idea is to use boosting to select out the available training data that are very distinguishable from the classified image data by automatically adjusting the weights of training instances. The remaining available data are treated as additional training data, which greatly boost the confidence of the learned model even when classified image training data are scarce. Support vector machine (SVM) that has been widely used for classification tasks in remote sensing image, are adopted in the classification validation experiments. The experiments are respectively carried out on Botswana Hyperion data set and University of Pavia data set including the same classes.

This paper is organized as follows: in Section 2, our transfer learning-based fusion method is described. Experimental results and discussion are present in Section 3 and conclusions are presented in Section 4.

## 2. Transfer Learning Based Fusion Method

**Figure 1:**
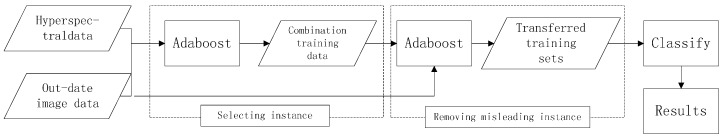
Framework of the classification scheme in this paper.

In this paper, we mainly use the instance-transferred idea and propose a method that combines transfer learning with data fusion. In many machine learning applications, the case exists that the training or labeled data are too sparse to train a classification model with better performance. In this case, the traditional learning methods require users to re-collect more labeled data, which is expensive in time and cost. However, there are often a lot of existing out-of-date data which are related to training data. Part of those data can be able to considered as the source domain and reused to instruct these problems as the target domain. Transfer learning can be applied in different learning domains. In transfer learning, we are particularly interested in transferring the knowledge from source task to a target task rather than learning all source and target tasks simultaneously, whereas, not all source instances are available to the target task. If the training sets in source data were sufficient, the algorithm efficiency would be lower due to excessive selection. To improve the efficiency of transfer learning, the framework of source instances based on adaboost has been carried out as shown in Figure 1. There are mainly two parts in this scheme, which include selecting instances and removing misleading instances.

### 2.1. Selecting the Source Domain Instances to Append Labeled Target Domain Instances

In transfer learning, the problem solved firstly is to implement the domain adaptation, one approach of which is instances transfer [16]. In this paper, we mainly use the instances transfer. However, not all source instances are available to the target task. If the source data are large scale, the algorithm efficiency would be lower because of the available selection. Thus, we select some source data firstly to implement preliminary domain adaptation. Generally, instances transfer methods are also motivated by instance weighting. In our work, we used the adaboost algorithm to select the source instances for transfer learning.

In this method, domain adaptation is firstly implemented. Generally, instances transfer methods will be motivated by instance weighting. The adaboost algorithm is used to select the source instances for transfer learning. The distribution of source domain distinguishes from the target domain; however, both domains are in the identical feature space. Assume the source domain instances set is Equation (1), and the target domain instances set is Equation (2):

(1) $$X^{S}=\left\{x_{1}^{S},x_{2}^{S},\cdots ,x_{m}^{S}\right\}$$

(2) $$X^{T}=\left\{x_{1}^{T},x_{2}^{T},\cdots ,x_{n}^{T}\right\}$$

If the source instances only instruct one type or some types, the labeled set is Equation (3), while the residual target labeled instance set is Equation (4). The preliminary selection scheme of source data is shown as follows:

(3) $$X^{T1}=\left\{x_{1}^{T1},x_{2}^{T1},\cdots ,x_{n1}^{T1}\right\},\;X^{T1}\subset X^{T}$$

(4) $$X^{T2}=\left\{x_{1}^{T2},x_{2}^{T2},\cdots ,x_{n2}^{T2}\right\},\;X^{T2}\subset X^{T},\;n_{1}+n_{2}=n$$

As it is expected that the source domain knowledge can instruct the target domain learning, the training procedure in the adaboosting is adopted to select the instances according to their weights. During training procedure of adaboosting, the weights of the instances wrongly classified are increased. The training dataset is composed of source instances and target labeled ones instructed. After training, those source instances with higher weights are similar to the target ones. The source instance set is *X^S^*, and the target instance-set *X^T^*^1^, where *n*_1_ < *m*. Let the labels of *X^S^* be 1, while the labels of *X^T^*^1^ are −1. In order to make two patterns instances of balance, *X^S^* can be divided into about *m*/*n*_1_ portions, that is Equation (5), where [.] is a rounding operator. Each portion of training datasets is Equation (6), which respectively train the classifiers based on adaboosting. The instances weights are updated during training, which of those instances wrongly classified increase. After a few rounds training, the source instances with higher weights that exceed a threshold *W* are considered that they have the similar property to the target domain. Then, they can be selected to form instances set $X_{sub}^{S}$.

(5) $$X^{S}=X_{1}^{S}\cup X_{2}^{S}\cup \cdots \cup X_{\left[m/n_{1}\right]}^{S}$$

(6) $$T_{i}=X_{i}^{S}\cup X^{T1},\;\left(i=1,2,\cdots ,[m/n_{1}]\right)$$

### 2.2. Removing “Misleading” Source Domain Instances

**Figure 2:**
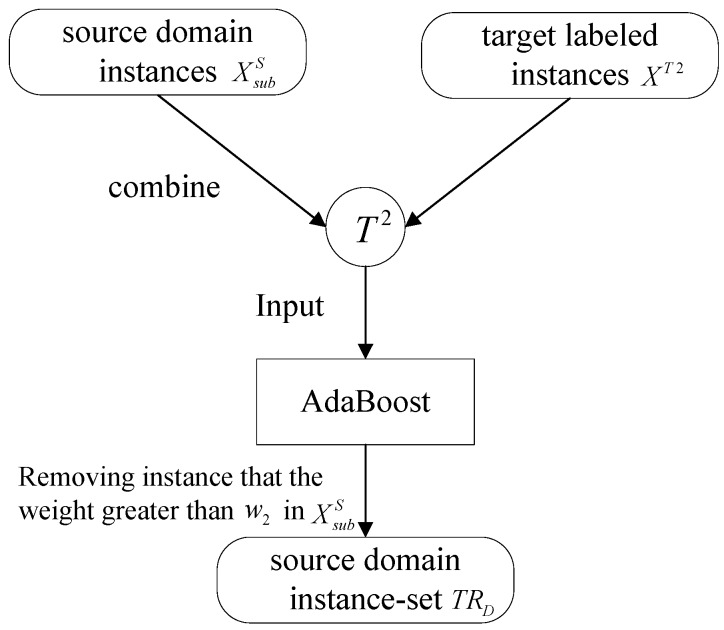
Framework of choosing the source domain instance-set.

However, the above method is only applied to select source instances for only one type of target domain. The source instances used to transfer learning should be easily classified from instances of other types. To avoid the negative transfer, adaboost is used to remove “misleading” source domain instances as shown in Figure 2. The training dataset is composed of $X_{sub}^{S}$ and the other types target labeled instance-set *X^T^*^2^. Unlike previous steps, we select the source instances classified correctly $X_{sub}^{S}$ from $X_{sub}^{S}$. $X_{sub1}^{S}$ is the final transfer instance-set to instruct the target learning.

The source domain instances selected out are as the training data with the labeled ones of target domain. In traditional classification model, the training instances are considered to have the same distributions as test instances. If the distributions varies, the traditional model is not acceptable and supposed to be modified.

The source domain instances selected out are as the training data with the labeled ones of the target domain. In traditional classification model, the distributions of the training and test instances are considered the same. When the distributions are different, the traditional model is not suitable and must be modified.

In the target domain, the instances set is *X^T^*, in which the unlabeled instances set is the test dataset $TS=\{x_{i}^{T})$, where $x_{i}^{T}\in X^{T}\left(i=1,2,\ldots ,k\right)$, and the labeled instances set whose distribution is similar to *TS* is denoted $TR_{S}=\left\{x_{i}^{T},y\left(x_{i}^{T}\right)\right\}$, where $x_{i}^{T}\in X^{T}\left(i=1,2,\ldots ,n\right)$, $y\left(x_{i}^{T}\right)$ is the label for $x_{i}^{T}$ and $TR_{S}=X^{T1}\cup X^{T2}$, so the size of *X^T^* is *k* + *n*. $X_{sub1}^{S}$ with a different distribution from *TS* is redefined as $TR_{D}=\left\{x_{i}^{S},y\left(x_{i}^{S}\right)\right\}$, where $x_{i}^{S}\in X^{S}\left(i=1,2,\ldots ,m\right)$. The training data is denoted as $TR=TR_{S}\cup TR_{D}$. Also $TR_{S}$ and $TR_{D}$ are respectively named the same-distribution dataset and the diff-distribution dataset. The scheme is as follows:

**Input:** Directed Network *N*; Nodes number *K*; Training rounds *T*; Sampling parameter *ρ*;

The target labeled instances set and the source instances on the *k*-th node are respectively $TR_{S_{k}}$ and $TR_{D_{k}}$, *k* = 1,2,…,*K*, where *l_S_*(*k*) is the size of $TR_{D_{k}}$;

**Initialize:** for any node *k* (*k* = 1, 2,…, *K*), the weight of instance *x_i_*:

(7) $$w_{k,t}(x_{i})=\{\begin{array}{cc} 1/l_{S}(k) & x_{i}\in TR_{S}\\ 1/l_{D}(k) & x_{i}\in TR_{D} \end{array}$$

**Do for:**

**Step 1.**  Generate a replicate training set $T_{k,t}$ of size $\rho l_{S}\left(k\right)+\rho l_{D}\left(k\right)$, by weighted sub-sampling with replacement from training set $TR_{S_{k}}$ and $TR_{D_{k}}$, *k* = 1, 2,…, *K*, respectively;
**Step 2.**  Train the classifier (node) $C_{k,t}$ in the classifiers network with respect to the weighted training set $T_{k,t}$ and obtain the hypothesis for multi-classification $h_{k,t}:x\mapsto Y$, *k* = 1, 2,…, *K*, where *Y* is the label set.
**Step 3.**  Calculate the weighted error rate of the instances in $TR_{S_{k}}$:
  (8) $$\varepsilon_{k,t}=\sum_{x_{i}\in TR_{S_{k}}}w_{k,t}(x_{i})I[y_{i}\neq h_{k,t}(x_{i})]$$
**Step 4.**  Hypothesize the classifier $C_{k,t}$ weight:
  (9) $$\alpha_{k,i}=0.5\times \log\left(\frac{1-\varepsilon_{k,i}}{\varepsilon_{k,i}}\right)$$
**Step 5.**  Set weights update parameters βk,i=εk,i1−εk,i, and γk=11+2ln(lD(k)/T. Note that *ε_k,t_* < 0.5.
**Step 6.**  Update the weight of instance *i* of node *k*:
  (10) $$\lambda_{k,i}(i)=-2\alpha_{k,i}(I-1/2)-2\sum_{n}\alpha_{n,i}(I-1/2),\;I[y(x_{i})\neq h_{k,i}(x_{i})]$$
  (11) $$w_{k,i+1}(x_{i})=\{\begin{array}{l} w_{k,i}(x_{i})\;\times \;\beta_{k,i}{}^{\lambda_{k,i}(i)},\\ w_{k,i}(x_{i})\;\times \;\gamma_{k}{}^{-\lambda_{k,i}(i)}, \end{array}\begin{array}{c} x_{i}\in TR_{S_{k}}\\ x_{i}\in TR_{D_{k}} \end{array}$$

where node *n* is neighbor of node *k*.

**Output:** Final hypothesis:

(12) $$H_{K,T}=\underset{y\in Y}{\arg\;\max}\sum_{k=1}^{K}\sum_{i=1}^{T}\left(\alpha_{k,i}\left[h_{k,i}(x)==y\right]+\sum_{n}\alpha_{n,i}\left[h_{n,i}(x)==y\right]\right)$$

As shown in the algorithm, in each round, the training subset is respectively sampled from $TR_{S}$ and $TR_{D}$ with the same sampling rate *ρ*. The hypothesis contains the classifier weight *α_k,t_*, which represents the classifier importance for the final hypothesis. It stabilizes the final result and tune to attain the stable classifier network. The weight update methods are different between the same-distribution instances and the diff-distribution instances. The weight update parameter of the same-distribution instances is $\beta_{k,t}$, while that of the diff-distribution ones is $\gamma_{k}$, which is related to the diff-distribution instances number on the current node. In each round, if a diff-distribution training instance is mistakenly predicted, it may likely conflict with the same-distribution training data. Then, adjustment should be introduced to its training weight to reduce its effect through multiplying its weight by $\gamma_{k}=\gamma_{k}{}^{-\lambda_{k,t\left(i\right)}}$, which is opposite to the same-distribution training data. After several rounds, the diff-distribution training instances with better fitting of the same-distribution will have larger training weights, while the dissimilar ones will have smaller weights.

## 3. Experiments and Discussion

In this section, we provide empirical evidence that incorporating the boosting algorithm into the knowledge transfer framework results in classification rate curves. We present results showing that our proposed method exhibits better classification rates than updating existing classifiers with data points selected either at random or via an existing, related general method. We also empirically show results that the proposed method offer a significant advantage over the more traditional semi-supervised methods by requiring far fewer data points to obtain better classification accuracies.

The proposed fusion classification method is tested on hyperspectral data sets obtained from two sites: NASA’s Okavango Delta, Botswana [26], University of Pavia and Pavia center [27]. Support Vector Machine (SVM) is selected for the basic learner.

### 3.1. Data Sets

#### 3.1.1. Okavango Delta, Botswana

The NASA EO-1 satellite acquired a sequence of data over the Okavango Delta, Botswana in 2001–2004. The Hyperion sensor on EO-1 acquires data at 30 m pixel resolution over a 7.7 km strip in 242 bands covering the 400–2500 nm portion of the spectrum in 10 nm windows. Preprocessing of the data was performed by the University of Texas Center for Space Research to mitigate the effects of bad detectors, interdetector miscalibration, and intermittent anomalies. Uncalibrated and noisy bands that cover water absorption features were removed, and the remaining 145 bands were included as candidate features: [10–55, 82–97, 102–119, 134–164, 187–220]. The data analyzed in this study, acquired 31 May 2001, consist of observations from 14 identified classes representing the land cover types in seasonal swamps, occasional swamps, and drier woodlands located in the distal portion of the delta. These classes were chosen to reflect the impact of flooding on vegetation in the study area. The class names and corresponding numbers of ground truth observation used in the experiments are listed in Table 1.

#### 3.1.2. ROSIS Data

The flight over the city of Pavia, Italy, was operated by the Deutschen Zentrum fur Luftund Raumfahrt (DLR, the German Aerospace Agency) in the framework of the HySens project, and managed and sponsored by the European Union. According to specifications, the number of bands of the ROSIS-3 sensor is 115 with a spectral coverage ranging from 0.43 to 0.86 μm. The data have been atmospherically corrected but not geometrically corrected. The spatial resolution is 1.3 m per pixel. Two data sets were used in the experiment.

##### University Area

**Figure 3:**
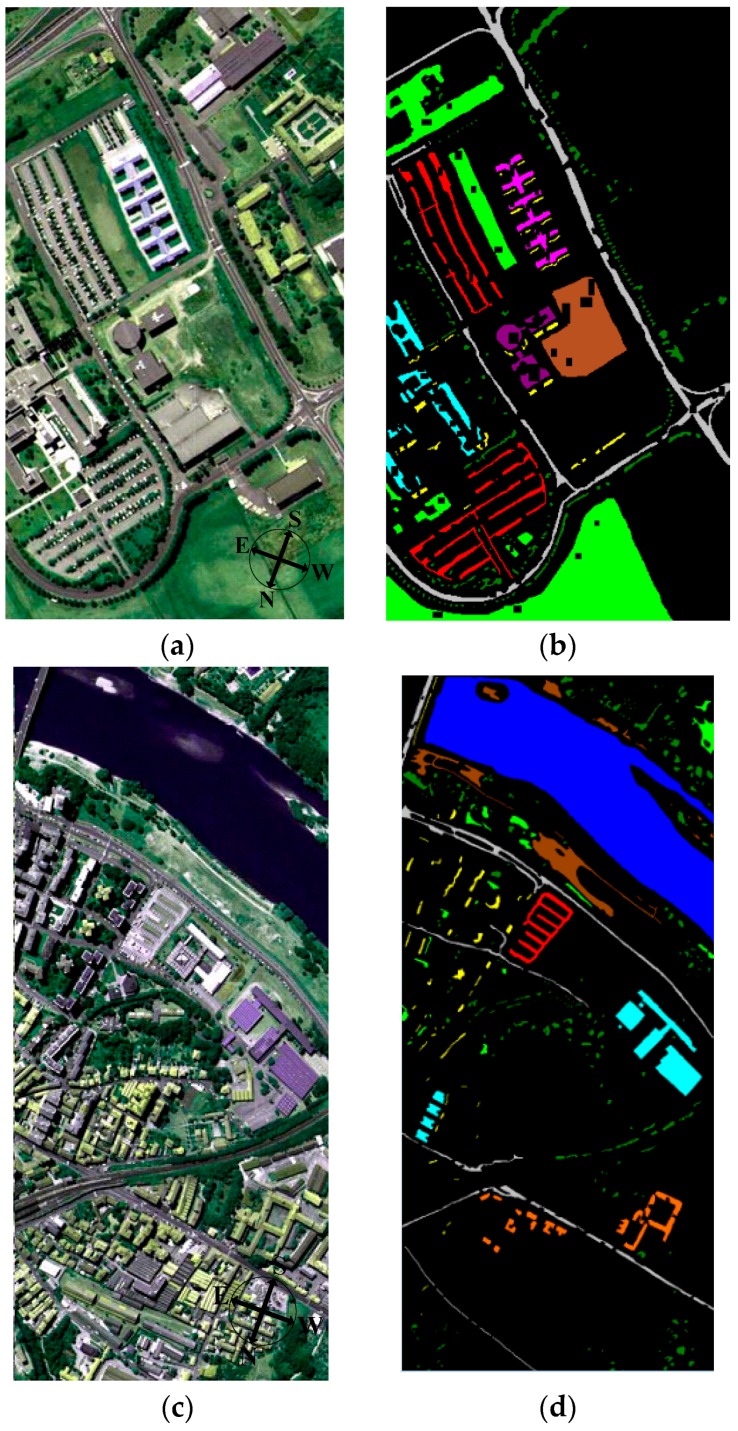
ROSIS data, three-channel color composite of the areas used for the classification: (**a**) University area; (**b**) Ground reference map of university; (**c**) Pavia center; (**d**) Ground reference map of center.

The first test set is around the Engineering School at the University of Pavia. The image is 610 *×* 340 pixels in size, with a spatial resolution of 1.3 m. The ROSIS sensor has 115 spectral channels, with a spectral range of 430–860 nm. The 12-noisiest channels were removed, and the remaining 103 spectral bands were used in this experiment. The reference data contain nine ground-cover classes: asphalt, meadows, gravel, trees, metal sheets, bare soil, bitumen, bricks, and shadows. This is a challenging classification scenario as the image is dominated by complex urban classes and spatially nested regions. True-color composite and related ground reference maps are shown in Figure 3, and the number of class-dependent labeled samples is shown in Table 2.

##### Pavia Center

The second test set is the center of Pavia. The Pavia center image was originally 1096 × 490 pixels. Some channels (13) have been removed due to noise. The remaining 102 spectral dimensions are processed. Nine classes of interest are considered, i.e., water, trees, meadows, bricks, soil, asphalt, bitumen, tiles, and shadows. Available training and testing set for each data set are given in Table 2, and Figure 3 shows false color images for both data sets.

### 3.2. Experiments

The assumption of transfer learning is that two data sets are different but related. It exploits relationships between data sets and extends a current statistical model to another data set. A class of popular transfer learning methods involves the updating strategy, whose origin is semi-supervised learning. Model parameters are updated by incorporating samples from the new data set. Therefore, a modified model can be generalized to the new data set.

In this section, we provide empirical evidence that incorporating adaboost into the knowledge transfer framework results in better accuracies. We present results showing that the proposed method exhibits better learning rates than traditional classifiers with data points selected by only stacking. We also empirically show results that this method have a significant advantage in few training samples, comparing with the more traditional methods, by requiring fewer data points to obtain better classification accuracies.

In these experiments, SVM was used as the basic learners in transfer adaboost. SVM classification (adopting the LIBSVM library [28]) was accomplished using a Gaussian RBF kernel. The SVM hyper parameters were optimized every ten iterations of the process by a fivefold cross validation. The C and *γ* parameters were selected in the range [2^−5^, 2^15^] and [2^−15^, 2^3^], respectively. In the experiment, the network structure selected is regular network with 20 nodes and the degree is 10, the sampling parameter *ρ* = 0.6, the training round *T* = 10. Furthermore, some constraints were added to the basic learners to avoid the case of training weights being unbalanced. Thus, during training procedure, the overall training weights between positive and negative examples are consistently balanced.

Three benchmark methods are implemented by using SVM as shown in Table 3. In the following, SVM, SVMt, TSVM will be used to represent various implementations of classifiers. SVMt means that training data will be only stacked by using SVM classifier. Moreover, the TSVM is the key method proposed in this paper.

**Figure 4:**
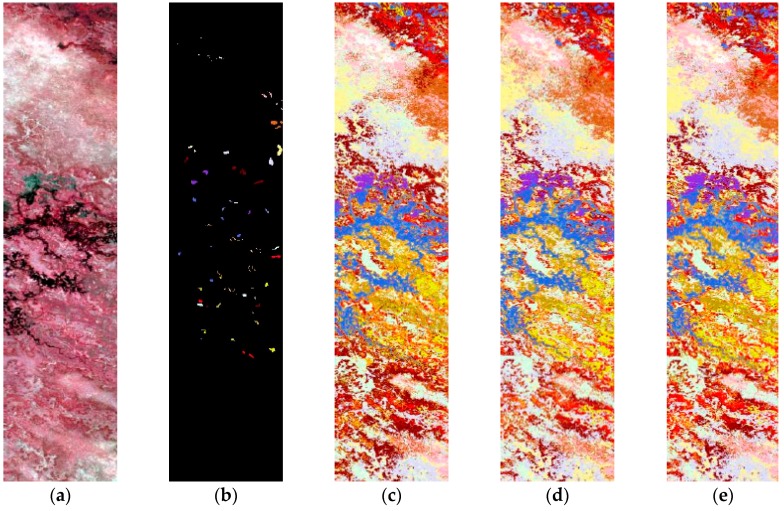
Classification maps achieved on the Botswana dataset. (**a**) RGB map; (**b**) Ground reference map; (**c**) SVM; (**d**) SVMt; (**e**) TSVM.

**Figure 5:**
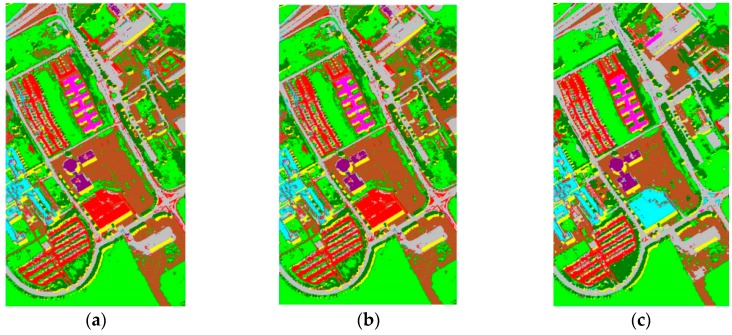
Classification maps achieved on the Pavia University dataset. (**a**) SVM; (**b**) SVMt; (**c**) TSVM.

The Botswana Hyperion data and ROSIS University of Pavia data set are respectively split into two sets: a training set *X^S^* and a test set *S*. We adopted KPCA algorithm to extract the image features including 30 dimensions [29]. The comparison experiment based on TrAdaBoost is performed. In the experiment, the network structure selected is regular network with 20 nodes and the degree is 10, the sampling parameter *ρ* = 0.6, the training round *T* = 10. Table 4 presents the experimental results of SVM, SVMt and TrAdaBoost (TSVM) when the ratio between training and testing data is 2% and 5%. The performance in classification accuracy was the average of 10 repeats by random. Finally, the Botswana data classification maps obtained by the different methods are shown in Figure 4 and the ROSIS data in Figure 5.

From Table 4, the accuracy given by TSVM are obviously higher than those given by SVM and SVMt. Intuitively, this is inevitable since SVM is not a learning technique designed for transfer classification, while adaboost is. However, as several researchers have already noted, transfer learning could not improve the generalization classification accuracy all the time and sometimes will show even lower performance on test set. This phenomenon is mentioned as transfer learning lowers the original performance negative transfer. Although in our experiments, adaboost continuously exhibit better or comparative performances than baselines, there is no guarantee for TrAdaboost to improve the basic learner.

**Figure 6:**
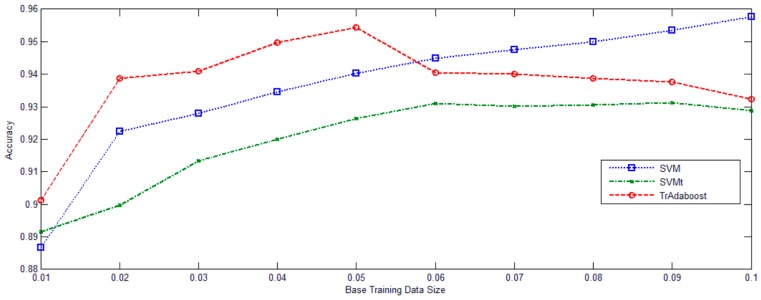
The accuracy curves on different ratios between training and testing.

In Figure 6, University of Pavia data set was deliberately used. The ratio between training and diff-distribution testing examples gradually increased from 0.01 to 0.1. Classifications were performed 10 times for each sampling rate. The average overall accuracies and standard deviations of the two baseline methods and the proposed method are listed in Figure 5. TrAdaBoost (SVM) consistently improves the performance of SVMt. TrAdaBoost (SVM) also outperforms SVM, when the ratio is lower than 0.05. But, when the ratio reaches larger than 0.05, TrAdaBoost (SVM) performs a little worse than SVM, but still comparative. Generally out-date image set training data contain both good knowledge and noisy data. In the case of that too few original image set training data could be used to train a good classifier, the useful knowledge from out-date image set training data will be beneficial to the learner, while the noisy part does not have significant negative effect.

In the following discussion, ROSIS data were used as an illustrative example. This data set combination is representative of the remaining data sets as due to its similarity with some classes. We first use SVM classifier on the University of Pavia image data set. The resultant graph presents a misleading clustering condition, and consequently leads to an unfaithful joint manifold. Subsequently, as seen in the example in Table 5, some misclassified samples are observed, e.g., for classes 2 and 4. It can be seen that the two classes, i.e., Meadow (Class 2) and Bare_soil (Class 4), exhibit significant confusion. Samples of Class 2 from the source image and samples of Class 4 from the target image are difficult to discriminate since the two features are very similar, which can also be validated by the confusion matrix in Table 4. The separation of these two classes is clearer in the latent space provided by the proposed method. The same trend is observed for Classes 3 and 4 of the data, as well as these two class pairs of the Class 3 and Class 2. The Asphalt (Class 1) and Brick (Class 6) land cover types also show some confusion.

In addition to improvement of the classification accuracy, the proposed method also selects the most informative data points from these classes. Compared to the results given by the two baselines, TSVM provides higher overall accuracy. Among the ten common classes in the UOP data pair, classes 1, 2, 3, 4, 6 are difficult to discriminate within a single image because the classes are comprised of mixtures. Spectral changes and mixed spectral signatures make domain adaptation in these data pairs even more difficult. The Class 2/Class 4 pair exhibits the most confusion. As shown in Figure 3, classes 2 and 4 from the source image (UOP) are very similar, and we can also observe that the spectral drifting of Class 2 is evident. Thus, many samples of Class 2 from the target image (COP) are misclassified as Class 4 when the training samples are only from the source image. The proposed method provides a significant improvement in the classification accuracy of Class 2. Table 6 shows the confusion matrix obtained by using the TSVM algorithm. This method eliminates the confusion among some classes, and also exhibits a better accuracy in tree (Class 3), bare_soil (Class 4), bitumen (Class 5) and shadow (Class 7).

## 4. Conclusions

In this paper, we have proposed a novel framework method for knowledge transfer fusion by boosting a basic learner. This algorithm is with high efficacy and accuracy and especially suitable to the small sample and similar classes discrimination. The basic idea is to select most useful instances as additional training data for predicting the labels. This method firstly finds the distribution of original image training data, and then selects the most helpful out-date image-training samples as additional training data. The methods, including SVMt and TSVM, show excellent performance as compared to SVM on two data sets (KSC and BOT). Our experiments on two hyperspectral data also demonstrate by using the method there will be a better transfer ability for similar classes discrimination than traditional learning techniques. The overall accuracy has been improved, and important is the most classes accuracies also have been improved. In addition to the concept level guidance, results show notable improvements especially for critical classes without scarifying much of the overall performance. TSVM further incorporates the informative analysis, thus performs the best.

Moreover, for case of small sample, this method exhibits a better performance than the benchmark methods. This study could be expanded when more hyperspectral data are available, especially to determine the effectiveness of the active learning based knowledge transfer framework when the spatial/temporal separation of the data sets is increased systematically.

## Figures and Tables

**Table 1:** Class names and number of data points for the Botswana data set.

No.	Class Name	Area 1	Area 2
1	Water	270	126
2	Hippo grass	101	162
3	Floodplain grasses1	251	158
4	Floodplain grasses2	215	165
5	Reeds1	269	168
6	Riparian	269	211
7	Firescar2	259	176
8	Island interior	203	154
9	Acacia woodlands	314	151
10	Acacia shrublands	248	190
11	Acacia grasslands	305	358
12	Short mopane	181	153
13	Mixed mopane	268	233
14	Exposed soils	95	89

**Table 2:** Information classes and true samples of COP and UOP.

No.	Center of Pavia	University of Pavia	COP	UOP
1	Asphalt	Asphalt	9248	6641
2	Meadow	Meadow	3090	18,649
3	Tree	Tree	7598	3064
4	Bare_soil	Bare_soil	6584	5029
5	Bitumen	Bitumen	7287	1330
6	Brick	Brick	2685	3682
7	Shadow	Shadow	2863	945
8	Tile	Gravel	42,826	2099
9	Water	Metal_sheet	65,971	1345

**Table 3:** The descriptions of baseline methods.

Benchmark	Training Data		Test Data	Basic Learner
	Labeled	Unlabeled		
SVM	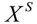	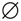	S	SVM
SVMt	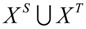	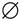	S	SVM
TSVM	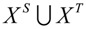	S	S	SVM

**Table 4:** The accuracy of three methods.

Ratio	Botswana			UOP		
	SVM	SVMt	TSVM	SVM	SVMt	TSVM
2%	0.9013	0.8832	0.9105	0.9225	0.8952	0.9387
5%	0.9171	0.9053	**0.9210**	0.9449	0.9265	**0.9543**

**Table 5:** Shows the confusion matrix obtained by the SVM method.

	Ground Truth (Pixels)								
	Class	Asphlt	Meadow	Tree	Bare_Soil	Bitumen	Brick	Shadow	
Classified image (pixels)	Asphalt	5953	19	1	23	303	205	6	
	Meadow	0	17,118	126	500	0	8	0	
	Tree	14	272	2937	40	0	2	0	
	Bare_soil	28	895	15	3867	0	44	0	
	Bitumen	218	0	0	0	1071	0	0	
	Brick	159	24	1	17	6	3476	2	
	Shadow	61	0	0	2	0	0	912	
	Accuracy	89.10	93.96	87.65	77.70	80.95	91.91	91.11	92.25

**Table 6:** Shows the averaged confusion matrix obtained by the TrAdaBoost (SVM) method.

	Ground Truth (Pixels)								
	Class	Asphalt	Meadow	Tree	Bare_Soil	Bitumen	Brick	Shadow	
Classified image (pixels)	Asphalt	5762	13	1	34	395	301	4	
	Meadow	3	16,546	193	601	0	6	0	
	Tree	14	207	3015	24	1	4	0	
	Bare_soil	17	526	12	4230	0	64	0	
	Bitumen	205	0	0	0	1080	4	0	
	Brick	129	33	0	61	58	3388	0	
	Shadow	25	0	1	0	0	0	949	
	Accuracy	88.57	93.21	89.82	85.07	83.69	89.49	95.41	93.87
